# Does chili pepper consumption affect BMI and obesity risk? A cross-sectional analysis

**DOI:** 10.3389/fnut.2024.1410256

**Published:** 2024-05-30

**Authors:** Mengxue Liu, Yihao Zhu, Fei Wang

**Affiliations:** ^1^Department of Anesthesiology, Sichuan Provincial People's Hospital, School of Medicine, University of Electronic Science and Technology of China, Chengdu, China; ^2^Department of Anesthesiology, Sichuan Clinical Research Center for Cancer, Sichuan Cancer Hospital & Institute, Sichuan Cancer Center, Affiliated Cancer Hospital of University of Electronic Science and Technology of China, Chengdu, China

**Keywords:** chili peper, body mass index (BMI), obesity, National Health and Nutrition Examination Survey (NHANES), weight management

## Abstract

**Background:**

The effects of chili intake on overweight and obesity have attracted significant interest in recent years. This study aimed to investigate the correlation between chili consumption frequency, body mass index (BMI), and obesity prevalence in the American population.

**Methods:**

Data from participants in National Health and Nutrition Examination Survey (NHANES) 2003–2006 were collected. We enrolled 6,138 participants with complete information on chili intake and BMI in this cross-sectional study. Multivariate logistic regression and sensitivity analyses were conducted to explore the relationship between chili intake frequency and BMI and obesity. Subgroup analyses and interaction tests were employed to assess the stability of the observed correlation.

**Results:**

Increased chili consumption frequency was linked to higher BMI values and a greater prevalence of obesity. Compared to the non-consumption group, the highest frequency group had a multivariate-adjusted β of 0.71 (95% CI: 0.05, 1.38) for BMI and an OR of 1.55 (95% CI: 1.22, 1.97) for obesity in the fully adjusted model. This positive association between chili intake frequency and obesity was more pronounced in females and older adults (≥ 60 years old).

**Conclusion:**

Our findings suggest a positive association between chili intake frequency and BMI and obesity in United States adults, suggesting that controlling chili intake frequency could potentially contribute to improved weight management in the general population.

## 1 Introduction

Obesity is a prevalent and financially burdensome chronic condition in the United States and around the world (1). Defined by body mass index (BMI) standards as a BMI ≥ 30 kg/m^2^ (2), obesity affects 35.0% of males and 40.4% of females in the United States, according to the National Health and Nutrition Examination Survey (NHANES) (3). This condition has been linked to various health risks, including reduced life expectancy, type 2 diabetes, cardiovascular diseases, specific cancers, kidney diseases, obstructive sleep apnea, gout, osteoarthritis, and liver and gallbladder diseases (4). To combat obesity, the Endocrine Society recommends achieving an energy balance through dietary control and increased physical activity as the most effective strategies (1, 4).

Historically, spices and herbs have been valued for their preservative, coloring, and flavor-enhancing properties (5). Among them, chili pepper stands out for its widespread use. Recent decades have seen a surge in research on the health benefits of chili peppers and their active ingredient, capsaicin. Interestingly, capsaicin has shown much promise in pain relief (6), cancer treatment (7, 8), cardiovascular health (9), lowering mortality rates (10), and even enhancing cognitive function in Alzheimer’s disease (11). Prior studies have demonstrated that chili peppers may decrease the prevalence of obesity by various mechanisms, including boosting energy expenditure (12), improving lipid oxidation (13), and decreasing appetite and energy intake (14). Furthermore, a prospective cohort analysis utilizing data from the China Health and Nutrition Survey (CHNS) revealed that although energy intake rises with greater chili pepper consumption, there exists a negative correlation between chili pepper intake and the occurrence of overweight/obesity (15). Nevertheless, a comprehensive cross-sectional study conducted on diabetes, obesity, and lifestyle in rural China revealed a significant association between the frequency of spicy food intake and overall obesity. These conflicting results underscore the necessity for additional investigation in this field (16). Furthermore, geographical and cultural differences lead to higher chili pepper consumption rates in Asian countries compared to Europe (17). This has resulted in a focus on Asian populations in most large-scale studies (15, 18–20). To date, the impact of chili pepper consumption on obesity in the American population, particularly in a large-scale study, has not been addressed. This study leverages NHANES data to investigate this relationship through a cross-sectional analysis of chili intake frequency, BMI, and obesity prevalence in the United States population.

## 2 Methods

### 2.1 Study population

**Figure 1:**
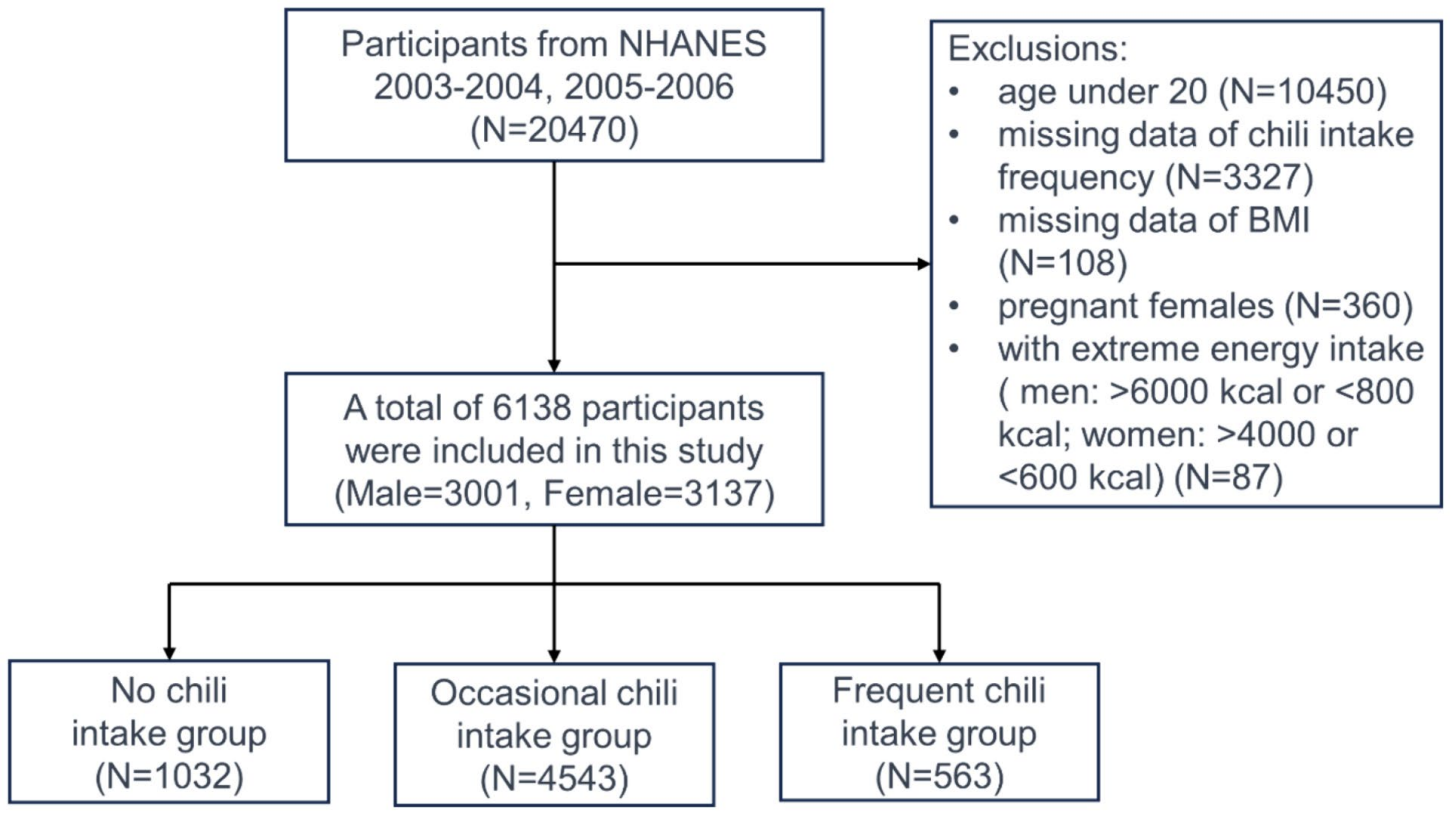
A flowchart for selecting participants.

Our study employed a cross-sectional approach which involved secondary data analysis from the National Health and Nutrition Examination Survey conducted by the National Center for Health Statistics (NCHS). NHANES aims to compile comprehensive demographic, health, and nutritional information across various age groups and ethnicities in the United States, outlining the incidence of significant diseases and their contributing factors. This data are crucial for national health and statistical purposes. Details on ethical considerations, consent procedures, and the survey’s methodology are available on the official NHANES website.^1^ For our analysis, we selected data for 20,470 participants from the 2003 to 2006 NHANES cycles. Participants excluded from the study were those under 20, with missing data on chili pepper consumption frequency and BMI, pregnant individuals, and those with implausible energy intakes (Figure 1).

### 2.2 Chili intake frequency

In this study, the exposure variable was participants’ chili consumption frequency over the preceding 12 months, as reported in their answers to the “How often did you eat chili?” question from the Food Frequency Questionnaire. According to their responses, we divided participants into three distinct groups: no chili intake group, occasional chili intake group, and frequent chili intake group. The groups correspond to a chili consumption frequency of zero times per month, less than once a week, and at least once a week, respectively.

### 2.3 Anthropometric measures

Weight and height were measured using established protocols and a standardized manual within Mobile Examination Centers (MECs). To determine BMI, we used the formula: weight in kilograms (kg) divided by height in meters squared (m^2^). Individuals with a BMI of 30 kg/m^2^ or higher were categorized as obese.

### 2.4 Covariates

To account for potential confounding variables associated with both chili consumption frequency and obesity, we incorporated several social demographic and lifestyle characteristics into the analysis as covariates. These included age, sex, race, educational attainment (categorized as below high school, high school graduate, and above high school), marital status (married, widowed, divorced/separated, and never married), family poverty income ratio (PIR, with values below 1 indicating poverty), smoking status (current smoker identified by a positive response to “Do you now smoke cigarettes?”), alcohol use (current drinker identified by an affirmative answer to “Have you had at least 12 alcoholic drinks in any form over the past year?”), physical activity level, diabetes status, and hypertension status (confirmed by a yes to “Have you ever been told by a doctor or other health professional that you had diabetes/hypertension?”).

National Health and Nutrition Examination Survey utilizes the Metabolic Equivalent of Task (MET) system to evaluate physical activity levels. This system assigns specific MET scores to various activities, reflecting the energy cost of each. The level of physical activity for each participant was calculated using the following equation: PA (MET-hours/week) = MET value × frequency of activity per week × duration of each activity session (21). Based on the calculated MET-hours per week, participants were categorized into three physical activity levels: those who were inactive, achieving less than 1 MET-hour per week; those engaging in low-intensity activity, ranging from 1 to 48 MET-hour per week; and those participating in high-intensity activity, exceeding 48 MET-hour per week. To accurately estimate habitual nutrient intake, NHANES collected dietary intake data over at least 2 non-consecutive days. The average intake of energy, proteins, carbohydrates, fats, sugars, and fibers was computed from these two-day dietary records.

### 2.5 Statistical analysis

The statistical analysis adhered to the guidelines set by the Centers for CDC, employing appropriate weighting measures. Continuous variables were presented as mean values along with their Standard Deviations (SDs), whereas categorical variables were expressed in counts and percentages. To evaluate differences among groups categorized by their chili consumption frequency, one-way ANOVA was used for continuous variables, and the Chi-Square test was utilized for categorical variables. The association between the frequency of chili consumption and BMI, as well as obesity, was investigated through multivariate linear regression for BMI as a continuous variable and logistic regression for obesity (BMI ≥ 30 Kg/m^2^) as a categorical variable, across three distinct models. The first model was unadjusted; the second model accounted for age, sex, and race; and the third model extended adjustments to include education, marital status, family PIR, smoking and drinking behaviors, physical activity levels, diabetes and hypertension status, and dietary intakes of energy, proteins, carbohydrates, fats, sugars, and fibers.

To explore potential variations in the association between chili consumption and BMI/obesity according to different participant characteristics, subgroup analyses were conducted. Stratification factors included sex, age (< 60/≥ 60 years), education attainment, family PIR (< 1/≥ 1), diabetes status, and hypertension status. These actors were also considered as pre-specified potential effect modifiers. Missing values for continuous variables were imputed using their median values, and K-Nearest Neighbors (K-NN) imputation was utilized for missing categorical data. All statistical analyses were conducted using statistical packages R (The R Foundation, version 3.4.3) and Empower (R) 2.0, with a significance threshold set at *p* < 0.05.

## 3 Results

### 3.1 Baseline characteristics

A total of 6,138 participants meeting inclusion criteria were enrolled in the study, with nearly equal representation from males (48.89%) and females (51.11%). Based on their reported chili consumption frequency, participants were divided into three groups: no chili intake (zero times per month), occasional chili intake (no more than once a week), and frequent chili intake (at least once a week). These groups comprised 16.81, 74.01, and 9.17% of the sample, respectively. In terms of lifestyle habits or risk factors potentially associated with obesity, the prevalence of smoking, alcohol consumption, hypertension, and diabetes among participants were 44.64, 69.78, 36.3, and 12.56%, respectively. The average BMI for the study population was 28.66 kg/m^2^, with 34.31% classified as obese.

Although no significant difference in BMI was observed among the three chili consumption groups (group 1: 28.30 ± 7.67; group 2: 28.70 ± 6.39; group 3: 28.99 ± 6.32, *p* = 0.099), a higher frequency of chili intake was associated with an increased prevalence of obesity (group 1: 29.65%; group 2: 34.95%; group 3: 37.66%, *p* = 0.001). Between the three chili consumption groups, significant differences were found in age, sex, ethnicity, education level, marital status, family PIR, alcohol consumption, physical activity level, diabetes status, and dietary intake (all *p* < 0.05). Smoking and hypertension rates did not differ significantly across the groups (all *p* > 0.05) (Table 1).

**Table 1:** Characteristics of the study population by frequency of chili intake.

Characteristics	No chili intake	Occasional chili intake	Frequent chili intake	*p*
	*N* = 1,032	*N* = 4,543	*N* = 563	
Age, years	53.59 (20.62)	51.88 (18.32)	49.91 (17.69)	< 0.001
Male	409 (39.63%)	2,275 (50.08%)	317 (56.31%)	< 0.001
Race/ethnicity				< 0.001
Mexican American	145 (14.05%)	623 (13.71%)	314 (55.77%)	
Other Hispanic	61 (5.91%)	84 (1.85%)	20 (3.55%)	
Non-Hispanic White	515 (49.90%)	2,800 (61.63%)	134 (23.80%)	
Non-Hispanic Black	249 (24.13%)	880 (19.37%)	67 (11.90%)	
Other races	62 (6.01%)	156 (3.43%)	28 (4.97%)	
Education				< 0.001
Below high school	319 (30.91%)	978 (21.53%)	257 (45.65%)	
High school	264 (25.58%)	1,206 (26.55%)	120 (21.31%)	
Above high school	449 (43.51%)	2,359 (51.93%)	186 (33.04%)	
Marital status				< 0.001
Married	509 (49.32%)	2,600 (57.23%)	337 (59.86%)	
Widowed	146 (14.15%)	445 (9.80%)	55 (9.77%)	
Divorced or separated	131 (12.69%)	595 (13.10%)	60 (10.66%)	
Never married	246 (23.84%)	903 (19.88%)	111 (19.72%)	
Family PIR	2.51 ± 1.50	2.85 ± 1.55	2.09 ± 1.38	< 0.001
Current smoker	435 (42.15%)	2034 (44.77%)	271 (48.13%)	0.067
Current drinker	641 (62.11%)	3,265 (71.87%)	377 (66.96%)	< 0.001
Physical activity				< 0.001
Inactive	469 (45.45%)	1769 (38.94%)	270 (47.96%)	
Low intensity	480 (46.51%)	2,384 (52.48%)	245 (43.52%)	
High intensity	83 (8.04%)	390 (8.58%)	48 (8.53%)	
Diabetes	129 (12.50%)	551 (12.13%)	91 (16.16%)	0.024
Hypertension	386 (37.40%)	1,651 (36.34%)	191 (33.93%)	0.383
Dietary intake				< 0.001
Energy intake, kcal/day	1866.14 (674.97)	2081.77 (761.72)	2093.02 (802.50)	
Protein intake, g/day	73.86 (30.21)	80.71 (32.27)	82.76 (35.67)	
Carbohydrate intake, g/day	233.59 (89.61)	253.28 (100.27)	264.81 (103.09)	
Fat intake, g/day	68.82 (31.52)	79.34 (35.02)	75.65 (35.60)	
Sugar intake, g/day	107.61 (57.14)	116.85 (62.89)	115.97 (59.84)	
Fiber intake, g/day	14.60 (7.36)	15.85 (7.65)	18.14 (9.32)	
BMI, Kg/m^2^	28.30 ± 7.67	28.70 ± 6.39	28.99 ± 6.32	0.099
Obesity	306 (29.65%)	1,588 (34.95%)	212 (37.66%)	0.001

Values are means (SDs) or *n* (%). Continuous variables were compared using one-way ANOVA. Categorical variables were compared with the chi-square test.

### 3.2 Chili intake frequency and increased body mass index values and obesity

Our research found a positive association between increased chili consumption and both higher BMI levels and a greater prevalence of obesity. When we analyzed chili consumption as a continuous variable in a fully adjusted model, we observed a small but statistically significant increase in BMI with more frequent chili intake (β = 0.08, 95% CI: −0.01, 0.16). Furthermore, analyzing chili consumption as categorical variables revealed similar trends. Individuals who frequently consumed chili had significantly higher average BMI figures compared to those who did not. Notably, the group with the most frequent chili consumption exhibited BMI values on average 0.71 units higher than those who abstained from chili (β = 0.71, 95% CI: 0.05, 1.38; *p*_trend_ = 0.015) (Table 2).

**Table 2:** Association between chili intake frequency and body mass index/obesity.

Chili intake frequency groups	BMI	Obesity
	β (95% CI)	OR (95% CI)
Crude model (model 1)		
Continuous	0.05 (−0.04, 0.14)	1.03 (1.00, 1.06)
Categories		
No Chili intake group	Reference	Reference
Occasional Chili intake group	0.40 (−0.05, 0.85)	1.27 (1.10, 1.48)
Frequent Chili intake group	0.69 (0.01, 1.37)	1.43 (1.15, 1.78)
*p* for trend	0.033	< 0.001
Minimally adjusted model (model 2)		
Continuous	0.08 (−0.01, 0.17)	1.04 (1.01, 1.07)
Categories		
No Chili intake group	Reference	Reference
Occasional Chili intake group	0.53 (0.08, 0.98)	1.34 (1.16, 1.56)
Frequent Chili intake group	0.81 (0.11, 1.50)	1.54 (1.22, 1.93)
*p* for trend	0.010	< 0.001
Fully adjusted model (model 3)		
Continuous	0.08 (−0.01, 0.16)	1.05 (1.01, 1.08)
Categories		
No Chili intake group	Reference	Reference
Occasional Chili intake group	0.50 (0.08, 0.93)	1.37 (1.17, 1.60)
Frequent Chili intake group	0.71 (0.05, 1.38)	1.55 (1.22, 1.97)
*p* for trend	0.015	< 0.001

In sensitivity analysis, the body mass index was converted from a continuous variable to a categorical variable (tertiles).95%CI, 95% Confidence interval; OR, Odds ratio; Model 1, No covariates were adjusted; Model 2, Adjusted for sex, age, and race; Model 3, Adjusted for sex, age, race, education level, marital status, family PIR, physical activity level, drinking and smoking status, diabetes, hypertension, and dietary data.

Our analysis also revealed a positive correlation between the frequency of chili consumption and obesity rates. In the fully adjusted model, an increase in chili consumption frequency (treated as a continuous variable) was associated with an increase in the odds of developing obesity (OR = 1.05, 95% CI: 1.01, 1.08). When we categorized chili consumption, those in the highest consumption group had a 55% greater risk of obesity compared to non-consumers (OR = 1.55, 95% CI: 1.22, 1.97; *p*_trend_ < 0.001) (Table 2).

### 3.3 Subgroup analysis

**Figure 2:**
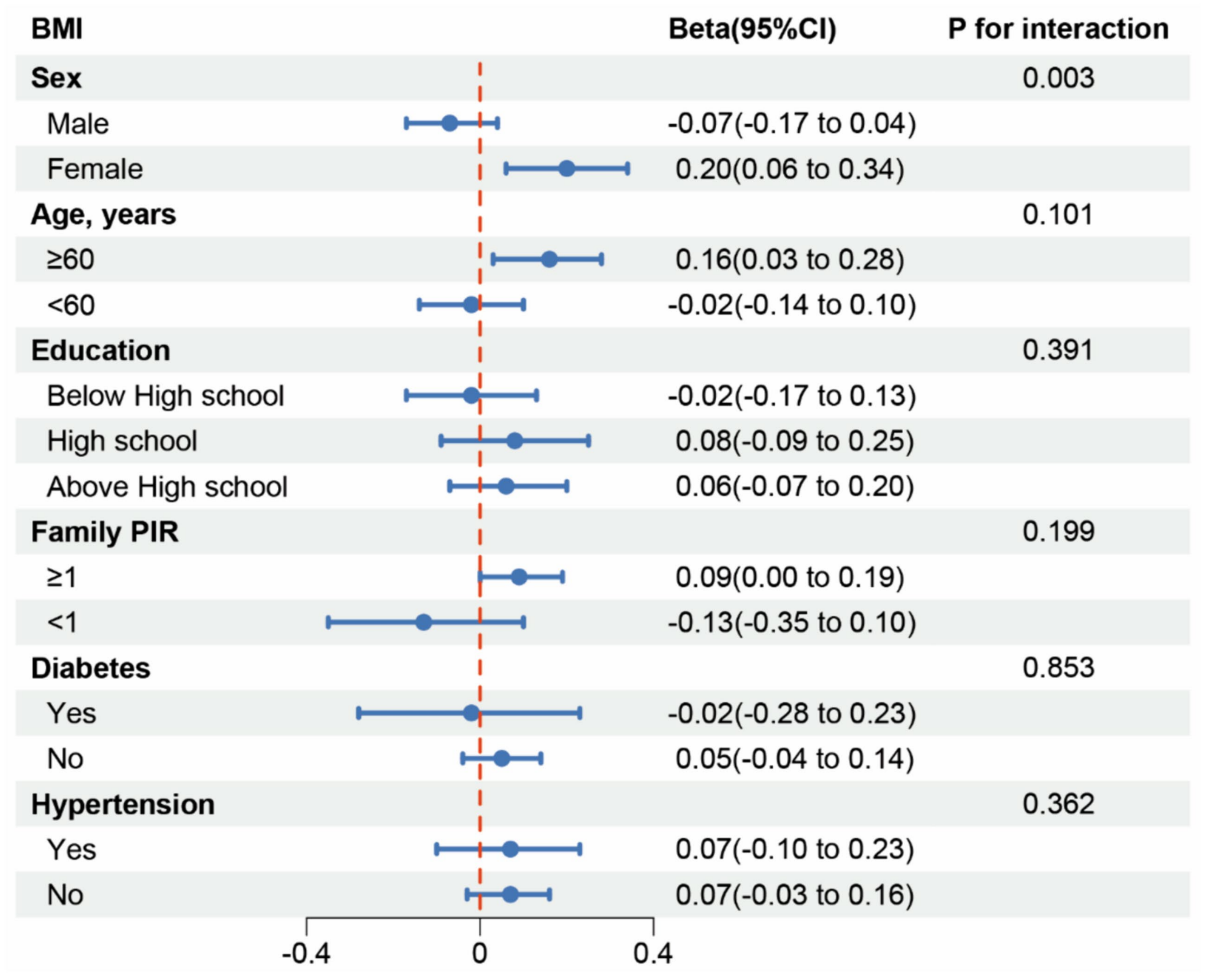
Subgroup analysis for the association between Chili intake and BMI.

In our study, subgroup analyses and interaction tests were conducted across various demographic factors, including age, sex, education level, family PIR, hypertension, and diabetes status, to explore how chili consumption frequency relates to both BMI and obesity. The findings revealed an inconsistent link, with interaction tests highlighting that only gender significantly modified the association between chili intake frequency and BMI (*p*_interaction_ = 0.003). Age, education level, family PIR, hypertension, and diabetes did not show significant interaction effects (all *p*_interaction_ > 0.05) (Figure 2).

**Figure 3:**
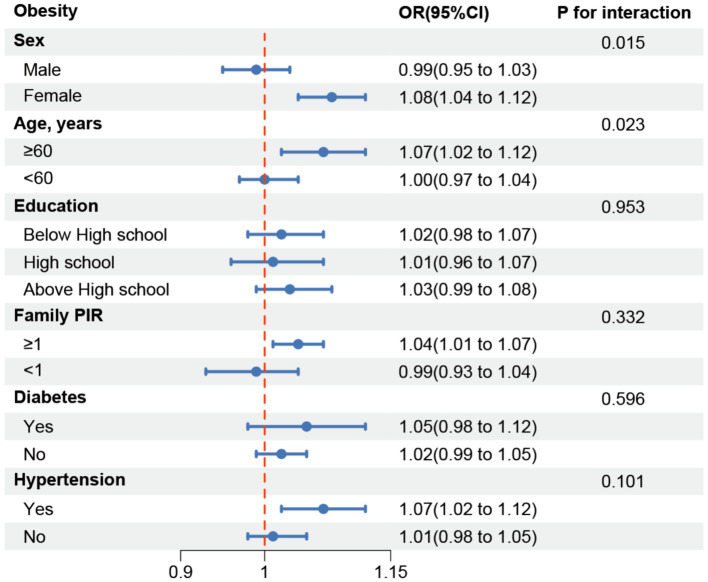
Subgroup analysis for the association between Chili intake and obesity.

Similarly, for obesity, significant interaction effects were found for sex and age (*p* for interaction = 0.015 and 0.023, respectively), suggesting that these factors influence how chili consumption frequency relates to obesity rates. Subgroup analyses indicated a positive association among women and individuals aged 60 and above. However, no significant interaction effects were observed for education level, family PIR, hypertension, and diabetes (all *p*_interaction_ > 0.05) (Figure 3).

## 4 Discussion

In this cross-sectional study of 6,138 participants, with 83.19% reporting a habit of chili intake, the overall obesity rate was 34.31%. We observed a positive correlation between chili consumption frequency and both BMI and obesity, with this correlation being more pronounced among female participants. These findings suggest that higher chili consumption frequency may be a risk factor for elevated BMI and obesity, particularly for women. Furthermore, controlling chili intake frequency may hold potential clinical value in weight management strategies.

To our knowledge, this is the first large-scale study to examine the association between chili pepper consumption frequency and both BMI and obesity in the American population. Overweight and obesity are increasingly serious non-communicable diseases, affecting over 100 million Americans or 68.5% of adults (22). Our findings of a positive correlation between chili pepper intake and both BMI and obesity, with this correlation being more pronounced among female participants, align with some large observational studies in Asian populations (16, 18). An epidemiological investigation encompassing 434,556 adults in China revealed that consumption of spicy foods poses a risk factor for obesity (19). This study found significant increases in covariate-adjusted BMI, adiposity, abdominal circumference, and waist-to-height ratios correlating with the frequency, intensity, and duration of spicy food consumption. Likewise, a meta-analysis focusing on the Chinese population indicated that individuals within the highest spicy food intake category had a higher risk of developing overweight/obesity conditions. Furthermore, subgroup analyses showed a significant dependence on this association in females (23). In contrast, several volunteer-based intervention studies have shown some potential benefits of chili pepper intake for weight control. For instance, Lee et al. conducted a 6-week randomized controlled study of chili pepper-containing sauce intake in a cohort of 60 patients and showed a reduction in waist circumference, cholesterol, and triglyceride levels in the chili pepper intake group (24). Additionally, findings from a 28-day randomized, double-blind, placebo-controlled trial involving 24 overweight but otherwise healthy subjects revealed that the intake of a novel food-grade slow-release formulation enriched with capsaicin led to reductions in both body weight and the waist-to-hip ratio (25). These contrasting findings might be related to the smaller sample sizes and shorter intervention periods of the intervention studies compared to our observational study. We found that in the stratified analysis of obese patients, the positive correlation appeared stronger in women, aligning with the findings of Yang et al. (16). They proposed that social factors, such as lack of medical resources and lower education levels among rural women, might explain this observation. However, the proportions of women with different education levels in our study (below High school, High school graduate, Above High school) were 49.1, 51.3, and 52.0%, respectively, with no statistical difference in the ratio of men to women. This suggests that the positive correlation between female sex and obesity may be related to biological factors, rather than the social factors proposed by Yang et al. Studies have shown that multiple genetic variants that significantly increase the risk of obesity only affect women, but not men (26).

Characteristics of the study population showed no statistically significant difference in the mean BMI across different chili intake frequency groups, whereas there was a difference in obesity rates (Table 1). Interestingly, multivariate regression analysis revealed that in the fully adjusted model, the BMI values and obesity rates in the highest chili intake frequency group were greater than those in the occasional and non-chili intake groups. This discrepancy suggests that these adjusted factors confound the differences in BMI among the groups. When exploring the relationship between chili consumption and obesity, it is necessary to consider that obesity is a multifactorial disease influenced by genetics, environment, diet, and lifestyle factors. Chili pepper intake is part of a dietary pattern that may be associated with higher overall calorie intake or unhealthy eating habits. For example, chili peppers are often consumed with high-fat, high-calorie foods (27). These dietary habits can lead to weight gain and partially explain why observational studies using spicy food intake as an exposure often find positive correlations, while intervention studies using capsaicin extract do not show significant changes in obesity indicators (28). Chili intake may impact potential weight gain by altering dietary patterns, and we need to determine the actual effect of this variable within a multifactorial context.

Research on the pharmacological mechanisms of capsaicin has yielded mixed results. Some studies suggest that chili pepper consumption contributes to increased lipid oxidation (12), activation of brown adipose tissue leading to heightened energy expenditure (29), suppression of energy intake through appetite and satiety regulation (30), and modulation of the gastrointestinal tract and intestinal microorganisms (31). However, other studies have shown no significant effect on energy expenditure and fat oxidation (32). In addition, excessive chili pepper intake can lead to reduced satiety and increased food intake (15). These contrasting findings highlight the need for further exploration of the exact mechanisms underlying the relationship between chili pepper intake and obesity.

This study utilized data from NHANES, a reliable and representative survey collected using multistage stratified probability sampling and standardized protocols. We adjusted for confounders and employed subgroup analyses and interaction tests to verify the robustness of the association between chili pepper intake frequency and both BMI and obesity. However, limitations of our study should be acknowledged. Firstly, the cross-sectional design precludes establishing a causal relationship between chili consumption frequency and BMI/obesity levels. Secondly, NHANES only collected data on chili pepper intake frequency for two cycles (2003–2004, 2004–2005) and lacked details on chili pepper type, spiciness, or intake amount, limiting our ability to examine these associations with BMI and obesity. Finally, despite adjusting for multiple covariates, it is highly conceivable that unmeasured patient factors influenced the results.

## 5 Conclusion

Our study identified a positive correlation between higher chili pepper intake frequency and both BMI and obesity prevalence. While these results suggest potential negative effects of frequent chili pepper consumption on weight management, it is essential to conduct well-designed clinical studies to determine the specific impact of chili pepper consumption. Future research should involve controlled clinical trials with appropriate and homogeneous populations, such as comparing the effects of chili pepper withdrawal in habitual consumers on various obesity-related variables. Such studies would help to dissect the specific effects of chili pepper and provide more definitive evidence regarding its role in weight management.

## Data availability statement

The datasets presented in this study can be found in online repositories. The names of the repository/repositories and accession number(s) can be found in the article/supplementary material.

## Ethics statement

Ethical approval was not required for the study involving humans in accordance with the local legislation and institutional requirements. Written informed consent to participate in this study was not required from the participants or the participants’ legal guardians/next of kin in accordance with the national legislation and the institutional requirements. The manuscript presents research on humans that do not require ethical approval for their study.

## Author contributions

ML: Data curation, Formal analysis, Software, Writing – original draft. YZ: Software, Visualization, Writing – review & editing. FW: Writing – review & editing.

## Footnotes

^1^ https://www.cdc.gov/nchs/nhanes/index.htm
